# Indole-3-carbinol synergistically sensitises ovarian cancer cells to bortezomib treatment

**DOI:** 10.1038/bjc.2011.546

**Published:** 2011-12-13

**Authors:** B Taylor-Harding, H Agadjanian, H Nassanian, S Kwon, X Guo, C Miller, B Y Karlan, S Orsulic, C S Walsh

**Affiliations:** 1 Women's Cancer Program and Division of Gynecologic Oncology, Burns and Allen Research Institute, Cedars-Sinai Medical Center, 8700 Beverly Boulevard, Los Angeles, CA 90048, USA; 2 Medical Genetics Institute, Cedars-Sinai Medical Center, 8700 Beverly Boulevard, Los Angeles, CA 90048, USA; 3 Department of Obstetrics and Gynecology, David Geffen School of Medicine, University of California at Los Angeles, Los Angeles, CA 90095, USA

**Keywords:** ovarian cancer, indole-3-carbinol (I3C), bortezomib, chemosensitivity, synergy, xenograft tumours

## Abstract

**Background:**

Bortezomib is a proteasome inhibitor with minimal clinical activity as a monotherapy in solid tumours, but its combination with other targeted therapies is being actively investigated as a way to increase its anticarcinogenic properties. Here, we evaluate the therapeutic potential of co-treatment with bortezomib and indole-3-carbinol (I3C), a natural compound found in cruciferous vegetables, in human ovarian cancer.

**Methods:**

We examined the effects of I3C, bortezomib and cisplatin in several human ovarian cancer cell lines. Synergy was determined using proliferation assays and isobologram analysis. Cell cycle and apoptotic effects were assessed by flow cytometry. The mechanism of I3C and bortezomib action was determined by RNA microarray studies, quantitative RT–PCR and western blotting. Antitumour activity of I3C and bortezomib was evaluated using an OVCAR5 xenograft mouse model.

**Results:**

I3C sensitised ovarian cancer cell lines to bortezomib treatment through potent synergistic mechanisms. Combination treatment with bortezomib and I3C led to profound cell cycle arrest and apoptosis as well as disruptions to multiple pathways, including those regulating endoplasmic reticulum stress, cytoskeleton, chemoresistance and carcinogen metabolism. Moreover, I3C and bortezomib co-treatment sensitised ovarian cancer cells to the standard chemotherapeutic agents, cisplatin and carboplatin. Importantly, *in vivo* studies demonstrated that co-treatment with I3C and bortezomib significantly inhibited tumour growth and reduced tumour weight compared with either drug alone.

**Conclusion:**

Together, these data provide a novel rationale for the clinical application of I3C and bortezomib in the treatment of ovarian cancer.

Ovarian cancer is the most lethal of gynaecologic malignancies, largely due to the late stage at diagnosis and development of chemoresistance after initial platinum- and paclitaxel-based combination chemotherapy. Treatment of patients with intrinsic or acquired chemoresistance represents a major clinical challenge (Bast *et al*, 2009). Furthermore, the molecular mechanisms underlying the aggressive biology of these tumours are poorly understood. This suggests that more effective therapeutic agents are needed to improve the treatment outcome of patients associated with biologically aggressive ovarian tumours, poor survival and chemoresistance (Etemadmoghadam *et al*, 2009; Nakayama *et al*, 2010). Strategies that overcome drug resistance and exploit pathways involved in tumourigenesis are attractive treatment options.

Bortezomib, the first-in-class proteasome inhibitor, has anticancer properties through wide-ranging mechanisms such as disruption of the cell cycle, promotion of apoptosis, and inhibition of proliferation and angiogenesis (Boccadoro *et al*, 2005). In both ovarian and colorectal tumour cell lines, bortezomib has been shown to inhibit cellular growth through upregulation of p27^kip1^ and induction of apoptosis (Uddin *et al*, 2008, 2009; Bruning *et al*, 2009), suggesting a possible therapeutic role for bortezomib in ovarian cancer. Several phase I clinical trials have evaluated the dose-limiting toxicities and maximum tolerated dose of bortezomib when combined with chemotherapeutic agents in ovarian cancer (Aghajanian *et al*, 2005; Cresta *et al*, 2008; Ramirez *et al*, 2008). However, a recent phase II study demonstrated minimal clinical activity of bortezomib as a single-agent treatment in recurrent platinum-sensitive ovarian or primary peritoneal cancer (Aghajanian *et al*, 2009). Currently, bortezomib is FDA approved and licensed for the treatment of multiple myeloma and mantle cell lymphoma, but it has generally not been an effective monotherapy in solid tumours. Combination of bortezomib with novel targeted agents has emerged as a treatment strategy that could broaden its clinical efficacy (Wright, 2010). We hypothesised that the combination of bortezomib with another agent could result in an effective treatment strategy for epithelial ovarian cancer.

Indole-3-carbinol (I3C) is a natural compound present in cruciferous vegetables, such as broccoli and cabbage. *In vitro* and *in vivo* studies demonstrate that I3C exhibits chemopreventive and anticancer properties in a variety of cancers, especially those that are hormonally responsive (Chinni *et al*, 2001; Rahman *et al*, 2006; Weng *et al*, 2008). Like bortezomib, I3C demonstrates anticarcinogenic properties through multiple mechanisms, including the induction of apoptosis, G1 cell cycle arrest, activation of the endoplasmic reticulum (ER) stress response and reversal of multi-drug resistance (Weng *et al*, 2008). Previous studies have demonstrated a potential benefit of I3C in the treatment of high-risk breast cancer, vulvar intraepithelial neoplasia and recurrent respiratory papillomatosis, while clinical trials of I3C are ongoing in cervical and prostate cancer (Rosen and Bryson, 2004; Reed *et al*, 2005; Naik *et al*, 2006). With the exception of a single study of I3C in human ovarian cancer cells (Raj *et al*, 2008), no further reports have investigated the biological effects nor clinical benefits of I3C in ovarian cancer.

Both I3C and bortezomib have been shown to target a broad spectrum of signalling pathways, which are likely to contribute to their ability to sensitise cells to apoptosis. Considering their potent anticarcinogenic properties and pleiotropic effects, we investigated the sensitivity of ovarian cancer cells and tumour xenografts to I3C and bortezomib combination treatment. In this report, we provide the first evidence that I3C and bortezomib work synergistically against ovarian cancer by promoting apoptosis, upregulating enzymes required for carcinogen metabolism, inducing ER stress, deregulating metabolic pathways, inhibiting carcinogenesis and reducing chemoresistance. These data provide support for the further investigation of I3C and bortezomib combination treatment in epithelial ovarian carcinoma.

## Materials and methods

### Cell culture and reagents

Human ovarian carcinoma cell lines OVCAR3, OVCAR5, OVCAR8, A2780, SKOV3, 3A, HEY and CAOV3 cells were purchased from American Type Culture Collection (Manassas, VA, USA). Cells were cultured in DMEM (MediaTech, Manassas, VA, USA) supplemented with 10% FBS, 100 U ml^−1^ penicillin and 100 *μ*g ml^−1^ streptomycin. Cell lines were cultured at 37°C and with 5% CO_2_ in a humidified atmosphere. Bortezomib (Velcade) was purchased from LC Laboratories (Woburn, MA, USA). Indole-3-carbinol, cisplatin and carboplatin were purchased from Sigma-Aldrich (St Louis, MO, USA). The following antibodies were used: RB, phospho-RB^S807/S811^, GADD45A, MET, SNAI1, CTBNN1, TOP2A and NFkB (Cell Signaling Technology, Danvers, MA, USA); CDK1, CYP1B1, p21^cip1^ and p27^kip1^ (Santa Cruz Biotechnology, Santa Cruz, CA, USA); ATF3 (Abcam, Cambridge, MA, USA); Actin (Sigma-Aldrich) and GAPDH (Fitzgerald, Acton, MA, USA). The following reagents and secondary antibodies were used in western blot analysis: Odyssey blocking buffer and Infrared IRDye-labelled secondary antibodies (LI-COR Biosciences, Lincoln, NE, USA).

### Proliferation assays

**Figure 1:**
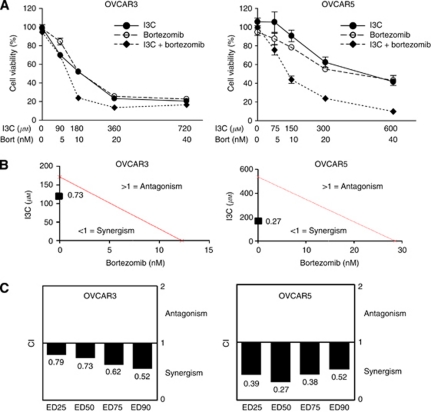
Indole-3-carbinol and bortezomib exhibit synergistic cytotoxicity in OVCAR3 and OVCAR5 cells. (**A**) Dose-dependent cytotoxicity of I3C, bortezomib and their combination. OVCAR3 and OVCAR5 cells were treated with I3C and bortezomib at the indicated concentrations for 48 h. Cell viability was measured as described in Materials and Methods. The data shown represent the mean±s.e.m. (*n*=3). (**B**) Isobologram analysis of combination I3C with bortezomib used in equipotent concentrations in OVCAR3 and OVCAR5 cells. The line designates the CI where CI=1 (additive effect). Combination index <1 indicates synergism and CI>1 represents antagonism. The combination data points (CI=0.73 for OVCAR3 and CI=0.27 for OVCAR5) calculated by CalcuSyn software indicate synergism at ED50. (**C**) The CI values of combination I3C and bortezomib at a range of EDs. TheCI values at ED25, ED50, ED75 and ED90 indicate a synergistic interaction between I3C and bortezomib in OVCAR3 and OVCAR5 cells.

**Figure 2:**
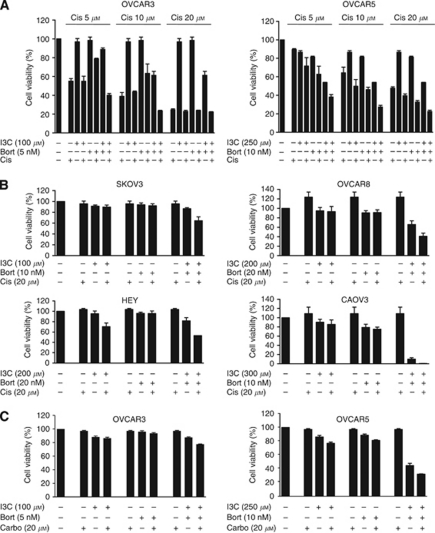
Indole-3-carbinol and bortezomib combination sensitises ovarian cancer cells to platinum-based chemotherapeutic agents. (**A**) OVCAR3 and OVCAR5 cells were treated with I3C, bortezomib, cisplatin or (**C**) carboplatin at the indicated concentrations for 48 h. Cell viability was measured as described in Materials and Methods. The data shown represent the mean±s.e.m. (*n*=3). (**B**) SKOV3, OVCAR8, HEY and CAOV3 cells were treated with I3C, bortezomib and cisplatin as in (**A**). The data shown represent the mean±s.e.m. (*n*=3).

Cells (4 × 10^3^ per 100 *μ*l per well) were plated in 96-well plates. After an overnight incubation, cells were treated with I3C, bortezomib, cisplatin or carboplatin at the indicated concentrations. After 48-h post-drug treatment, MTS/PMS solution (MTT Assay, Promega, Madison, WI, USA) was added according to the manufacturer's instructions and the absorbance recorded at 490 nm on an Ultramark-EX Microplate spectrophotometer (BioRad, Hercules, CA, USA) (Figure 1). For cisplatin and carboplatin experiments (Figure 2), we used a luminescent-based assay (CellTiter-Glo Luminescent Cell Viability Assay, Promega) for increased sensitivity. After 48-h post-drug treatment, CellTiter-Glo reagent was added to each well according to the manufacturer's protocol. Luminescence was measured on a Veritas microplate luminometer (Turner BioSystems, Sunnyvale, CA, USA) after 15 min. IC50 values were determined and used for the evaluation of drug interactions. Multiple independent experiments were performed in triplicate, and data were expressed as a relative percentage compared with the untreated control group set at 100%.

### Flow cytometry

Cells (5 × 10^5^) were plated into 100-mm tissue culture plates. After an overnight incubation, I3C and bortezomib were added at the indicated concentrations and samples were harvested 24 h post treatment. For cell cycle analysis, samples were fixed in 70% ethanol and subsequently treated with propidium iodide (PI) (Sigma-Aldrich) and RNase A (Sigma-Aldrich). Samples were analysed for PI incorporation with a Becton Dickinson FACScan (Franklin Lakes, NJ, USA) using ModFit LT software (Verity Software House, Topsham, ME, USA). For the apoptosis assay, cells were co-stained with annexin V and PI according to the manufacturer's protocol (Annexin V-FITC Apoptosis Detection Kit, BD Biosciences, San Jose, CA, USA), followed by flow cytometric analysis using CellQuest version 3.1 software (BD Biosciences) to gate viable, early and late apoptotic cells. The results generated were from multiple independent experiments performed in triplicate. A total of 10 000 events were collected for final analysis.

### Isobologram analysis

Synergy between I3C and bortezomib was studied as previously described (Taylor-Harding *et al*, 2010). To calculate the combined effects of the drugs, the combination index (CI) isobologram method was used (Chou and Talalay, 1984). Assessment of synergy was performed using CalcuSyn software (Biosoft, Cambridge, UK). Combination index values <1, =1 and >1 indicate synergy, additivity and antagonism, respectively.

### Western blot analysis

Western blot was performed as previously described (Taylor-Harding *et al*, 2010) with the following modifications. The membrane was blocked for 1 h with Odyssey blocking buffer (LI-COR Biosciences) and immunoblotted with primary antibody overnight at 4°C in blocking buffer supplemented with 0.1% Tween-20. After washing in TBST (0.1% Tween-20 in TBS), the membrane was probed with Infrared IRDye-labelled secondary antibody (LI-COR Biosciences) (1:10 000 dilution) in blocking buffer with 0.1% Tween-20 for 1 h. After washing, the membrane was visualised using the Odyssey Infrared Imager (LI-COR Biosciences).

### Quantitative RT–PCR (qRT–PCR)

Total RNA was isolated with TriReagent (Molecular Research Center, Cincinnati, OH, USA) and purified with the RNeasy kit (Qiagen, Germantown, MD, USA). Complementary DNA was generated by reverse transcription with Superscript III (Invitrogen, Carlsbad, CA, USA) and oligo dT priming. Quantitative RT–PCR was performed on the BioRad iCycler using QuantiTect SYBR Green (BioRad) according to the manufacturer's protocol. The results generated were from two independent experiments performed in triplicate. Quantitation was calculated by the comparative method (2^−ΔΔCt^) and data expressed relative to cells treated with vehicle (mock) set to 1. Primer sequences used in qRT–PCR are listed in Supplementary Table S1.

### Microarray gene expression profiling

OVCAR5 cells were treated with vehicle, 37.5 nM bortezomib, 675 *μ*M I3C, or with I3C and bortezomib in combination for 24 h. Three independent experiments were performed for a total of four triplicate conditions (12 samples). Total RNA was isolated as described for qRT–PCR analysis and the quality of RNA confirmed using an Agilent 2100 bioanalyzer (Santa Clara, CA, USA). Probe labelling, microarray hybridisation, washing and scanning were carried out as per manufacturer's instructions (Illumina, San Diego, CA, USA). Twelve samples were used to probe the Illumina HumanHT-12 v4 Expression BeadChip containing 47 231 human gene transcripts.

### Microarray data normalisation and analysis

Quality control of the microarray expression data was performed using the lumi R package (Du *et al*, 2008). We applied the lumiT function with a default setting to check the assumption of a constant variance. Variance-stabilising transformation was performed when needed. To remove systematic variation of non-biological origin, the data were normalised via the lumiN function, which performs quantile normalisation. Quality control of the normalised data was performed using the lumiQ function in the lumi R package. To identify differentially expressed genes, the cleaned data were analysed using the eBayes function in limma R package (Smyth, 2005). An empirical Bayes method to shrink the probe-wise sample variances towards a common value was utilised. Genes that had FDR adjusted *P*-values <0.01 were selected as differentially expressed. Among these, the top 1000 significantly altered genes (*P*<0.0025) were analysed. The selected genes were then compared with the Gene Ontology (GO) database using the Fisher's exact test, assuming a hypergeometric distribution, to evaluate gene set enrichment. The GOstats R package (Falcon and Gentleman, 2007) was utilised for the gene set enrichment analysis.

### *In vivo* tumour xenograft studies

Six-week-old female nude mice were obtained from Charles River Laboratories (Wilmington, MA, USA) and maintained according to IACUC guidelines. Mice were inoculated subcutaneously in both flanks with an equal volume of 8 × 10^6^ OVCAR5 cells and matrigel (Becton Dickinson) in a total volume of 200 *μ*l. Mice were randomly divided into four treatment groups with four mice per group (eight tumours). Treatments were as follows: vehicle (control); I3C alone (20 mg kg^−1^); bortezomib alone (1 mg kg^−1^) and the drug combination (20 mg kg^−1^ I3C with 1 mg kg^−1^ bortezomib). Treatment was given intraperitoneally twice weekly starting 4 days post inoculation. Tumour size was measured twice weekly with a caliper, and tumour volume was calculated as follows: *L* × *W*^2^, where *L*=length and *W*=width. Data were expressed relative to the initial tumour volume 4 days post inoculation. The initial tumour volume was set to 1 for each treatment group.

### Statistical analysis

Data were analysed using a two-tailed Student's *t*-test. A *P*-value of <0.05 was considered statistically significant.

## Results

### Synergistic cytotoxicity between I3C and bortezomib

To determine whether I3C and bortezomib exhibit a combined effect in ovarian cancer cells, we examined the effect of individual and combination treatment with I3C and bortezomib after 48-h exposure using the MTT assay. To ensure that the contributing effects from each drug was equivalent, the IC50 for each drug was determined and serial dilutions were generated based on the IC50 of each drug providing a 1:1 equipotent I3C/bortezomib ratio. We found that the drug combination caused greater inhibition of cellular proliferation than either drug alone in OVCAR3 and OVCAR5 cells (Figure 1A), an effect that was reproducible in a panel of ovarian cancer cell lines (Figure 2B and data not shown). To determine whether I3C and bortezomib interact synergistically, isobologram analysis was performed. This analysis provides a CI value that measures the degree of interaction between two or more drugs, where a CI <1 and a CI >1 indicates synergism and antagonism, respectively (Chou and Talalay, 1984). A CI of 0.73 and 0.27 was identified for OVCAR3 and OVCAR5 cells, respectively, when the effective dose (ED) of both agents inhibited cell viability by 50% (Figure 1B). Irrespective of high cytotoxicity (90% inhibition, ED90) or low cytotoxicity (25% inhibition, ED25), the CI values remained below 1, indicating that synergism occurs independently of the equipotency levels of I3C and bortezomib (Figure 1C). Our data demonstrate that I3C and bortezomib exhibit a robust synergistic interaction in ovarian cancer cells, particularly in OVCAR5 cells.

### I3C and bortezomib combination sensitises ovarian cancer cells to standard platinum-based chemotherapeutic agents

To determine whether I3C and bortezomib could sensitise cells to cisplatin, we treated OVCAR3 and OVCAR5 cells with subtoxic doses of equipotent I3C and bortezomib with increasing concentrations of cisplatin. We found that combination I3C and bortezomib could sensitise cells to cisplatin in a dose-dependent manner (Figure 2A). To ensure that this effect was not limited to these cell lines, we tested a panel of ovarian cancer cell lines with equipotent concentrations of I3C and bortezomib plus cisplatin. Interestingly, we found that in all six cell lines tested, co-treatment with I3C and bortezomib conferred sensitivity to cisplatin (Figure 2B and data not shown). To further explore the cytotoxic effects of I3C and bortezomib with conventional platinum-based chemotherapeutics, we examined the effect of carboplatin with I3C and bortezomib in OVCAR3 and OVCAR5 cells. Similarly, co-treatment with I3C and bortezomib could sensitise cells to carboplatin (Figure 2C) suggesting that I3C and bortezomib can increase the antitumour effects of standard platinum-based chemotherapeutic agents.

### I3C and bortezomib combination enhances apoptosis

**Figure 3:**
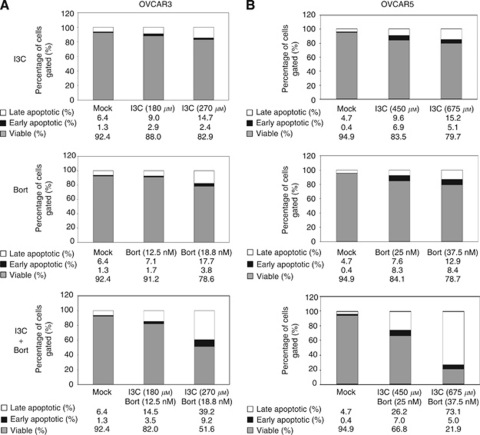
Indole-3-carbinol and bortezomib enhance apoptosis in OVCAR3 and OVCAR5 cells. (**A**) OVCAR3 and (**B**) OVCAR5 cells were treated with I3C, bortezomib or their combination for 24 h at the indicated concentrations. The cells were subsequently co-stained with annexin V and PI, and viable (annexin V-negative and PI-negative), early apoptotic (annexin V-positive and PI-negative) and late apoptotic (annexin V-positive and PI-positive) cells were distinguished by flow cytometric analysis. The stacked bar graph represents the mean percentage of viable (grey), early apoptotic (black) and late (white) apoptotic cells from a triplicate experiment.

To determine whether the cytotoxic effects of I3C and bortezomib were due to apoptosis, annexin V and PI co-staining was performed followed by flow cytometric analysis. As the cytotoxic effects of I3C and bortezomib were evident at 24 h, we determined the effects on apoptosis at this time point to assess for direct effects. To evaluate the apoptotic effects of equipotent doses of I3C and bortezomib, the IC10 for each drug was established for OVCAR3 and OVCAR5 cells, which provided an initial concentration that caused minimal (10%) cytotoxicity. The IC10 for I3C in OVCAR3 and OVCAR5 were 180 and 450 *μ*M, respectively, which induced apoptosis in both cell lines (Figure 3, upper panels). Raising the IC10 concentration by 1.5 fold increased apoptosis in a dose-dependent manner. The IC10 for bortezomib in OVCAR3 and OVCAR5 cells were 12.5 and 25 nM, respectively. Increasing concentrations of bortezomib similarly induced apoptosis in a dose-dependent manner (Figure 3, middle panels). When OVCAR3 and OVCAR5 cells were treated with combination I3C and bortezomib, a dose-dependent increase in apoptosis was observed compared with using either drug alone (Figure 3, lower panels). Specifically, we found that OVCAR3 cells treated with maximum doses of I3C/bortezomib increased apoptosis by 31.3% and 26.9% compared with I3C or bortezomib alone, respectively. OVCAR5 cells treated with maximum doses of I3C/bortezomib increased apoptosis by 57.8% and 56.8% compared with I3C or bortezomib alone, respectively. These data indicate that I3C and bortezomib induce apoptosis, and that the drug combination significantly enhances this effect, especially in OVCAR5 cells.

### I3C and bortezomib combination induces cell cycle arrest

**Figure 4:**
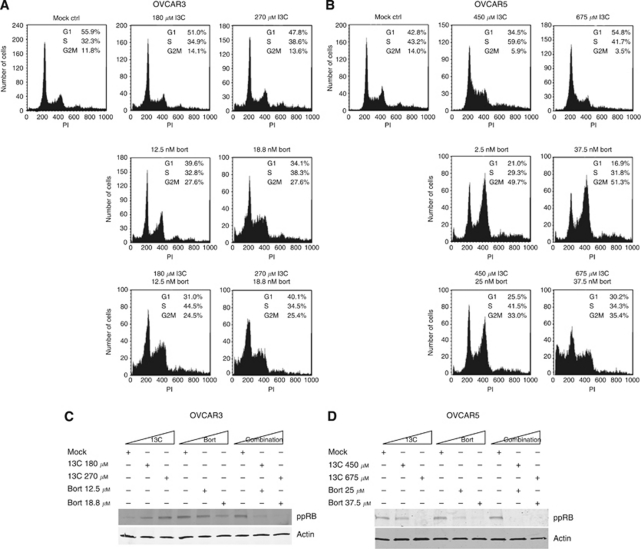
Indole-3-carbinol and bortezomib cause cell cycle arrest in OVCAR3 and OVCAR5 cells. (**A**) OVCAR3 and (**B**) OVCAR5 cells were treated with I3C, bortezomib or in combination for 24 h at the indicated concentrations. Cells were fixed and stained with PI followed by flow cytometric analysis for DNA content as described in Materials and Methods. A representative DNA histogram is shown for each condition. The mean percentage of cells from a triplicate experiment is indicated for cells in G1-, S- and G2-M-phase for each condition. (**C**) OVCAR3 and (**D**) OVCAR5 cells were treated with I3C, bortezomib or in combination as in (**A**) and (**B**), respectively. Whole-cell extracts were isolated and immunoblotted with the indicated antibodies. Actin was used as a loading control.

To determine whether the cytotoxic effects of I3C and bortezomib could be attributed to alterations in the cell cycle, OVCAR3 and OVCAR5 cells were treated with I3C and bortezomib for 24 h and subjected to PI flow cytometric analysis. Identical drug concentrations used for the annexin V/PI apoptosis assay were used for cell cycle analysis. We found that in OVCAR3 cells, increasing concentrations of I3C reduced the percentage of cells in G1 (Figure 4A), whereas I3C induced a G1 arrest at maximal concentration (675 *μ*M) in OVCAR5 cells (Figure 4B). Increasing bortezomib concentrations induced a G2-M arrest in both OVCAR3 and OVCAR5 cells, although this effect was more pronounced in OVCAR5 cells (Figures 4A and B). In both OVCAR3 and OVCAR5 cells, co-treatment with I3C and bortezomib shifted the cell cycle towards G2-M in a dose-dependent manner (Figures 4A and B). Additionally, OVCAR5 cells treated with combination I3C and bortezomib at maximum concentration exhibited a significant sub-G1 peak indicative of apoptosis (Figure 4B, bottom right), consistent with the elevated levels of apoptosis observed in annexin V/PI assays (Figure 3B, bottom right). These data suggest that while individual treatment with I3C and bortezomib have different effects on cell cycle distribution that appear to be context-dependent, the combination of these agents commonly impede the cell cycle by exerting a G2-M arrest.

In parallel, we sought to determine whether co-treatment with I3C and bortezomib may elicit a cell cycle arrest at the G1-S boundary. Co-treatment with increasing I3C and bortezomib severely reduced the levels of phosphorylated RB compared with using either drug alone (Figures 4C and D). This suggests that I3C and bortezomib can prevent RB phosphorylation and maintain RB in an active repressive state thereby blocking the cell cycle at the G1-S transition.

### I3C and bortezomib combination affects multiple pathways important for cancer progression

**Figure 5:**
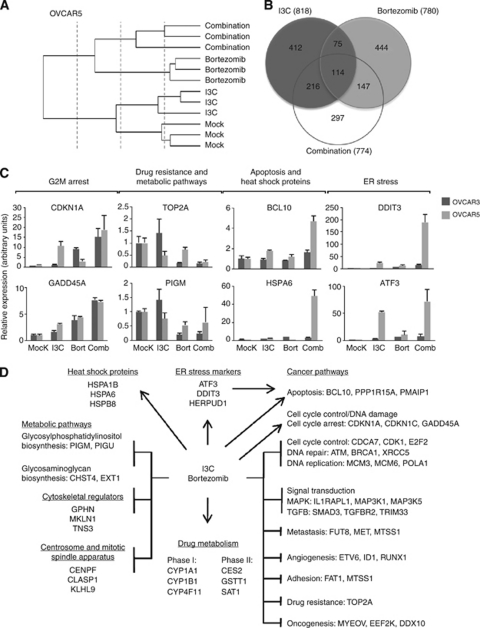
Indole-3-carbinol and bortezomib combination inhibits carcinogenesis, reduces chemoresistance, upregulates ER stress markers, deregulates metabolic pathways and causes widespread gene deregulation in OVCAR3 and OVCAR5 cells. OVCAR5 cells were treated with vehicle (mock), 675 *μ*M I3C, 37.5 nM bortezomib or in combination for 24 h with subsequent RNA isolation and microarray gene expression analysis. (**A**) A dendrogram presentation of the data from triplicate experiments indicate that replicate samples cluster together. (**B**) Venn diagram representing overlap between I3C and bortezomib treatment. Target genes with log-fold changes >0.4 or <−0.4 (*P*<0.0025) are presented. (**C**) Quantitative RT–PCR of candidate target genes identified by microarray analysis categorised by function. Target gene validation was also performed in OVCAR3 cells treated with vehicle (mock), 270 *μ*M I3C, 18.8 nM bortezomib or in combination for 24 h. (**D**) Pleiotropic effect of I3C and bortezomib on multiple biological processes. Representative target genes with log-fold changes >1.5 or <−1.5 (*P*<0.0025) are shown.

To determine the mechanism responsible for the synergistic effect of I3C and bortezomib, we performed RNA microarray analysis. Considering that both the apoptotic and synergistic effects of I3C and bortezomib were more robust in OVCAR5 cells compared with OVCAR3 cells at equipotent doses, we selected OVCAR5 cells for microarray analysis. We treated OVCAR5 cells with vehicle (mock), 675 *μ*M I3C, 37.5 nM bortezomib or combination for 24 h, identical to the maximum concentrations used for our apoptosis and cell cycle studies. Subsequent microarray analysis of replicate samples from triplicate experiments shared similar gene expression patterns that clustered together in the dendrogram (Figure 5A) demonstrating the high reproducibility of our results.

We focused on significantly altered genes (*P*<0.0025) with log-fold changes >1.5 (upregulated) or <−1.5 (downregulated). While I3C treatment has significantly more differentially expressed genes (216 genes) in common with co-treatment compared with bortezomib (147 genes), the majority is unique to the combination condition (297 genes) (Figure 5B). In total, I3C/bortezomib treatment altered the expression of 774 genes. Classification of these genes indicate that co-treatment with I3C and bortezomib induces gene expression changes in multiple pathways, particularly carcinogenesis (Supplementary Table S2), consistent with the GO gene enrichment dataset (data not shown). Validation of our microarray data by qRT–PCR and western blot analysis in both OVCAR3 and OVCAR5 cells showed that target genes involved in cell cycle control (e.g., *CDKN1A* and *CDK1*), apoptosis (e.g., *BCL2L1* and *BCL10*) and signal transduction (e.g., *DUSP1* and *NFkBIB*) were significantly deregulated (Figure 5C, Supplementary Figure S1 and data not shown). Moreover, metastasis (e.g., *MET* and *SNAI1*), angiogenesis and adhesion target genes showed altered expression (Supplementary Figure S1 and Supplementary Table S2). Notably, co-treatment with I3C and bortezomib appeared to downregulate *TOP2A* and *ABCC4*, target genes that are typically associated with chemoresistance. Consistent with our microarray data, qRT–PCR showed that *TOP2A* was severely downregulated (Figure 5C), a result that was reproducible by western analysis in OVCAR3 cells but not in OVCAR5 cells (Supplementary Figure S1B), suggesting that these effects are transient and/or evident only at the transcriptional level.

Besides promoting cell death and inhibiting cancer progression, the combination of I3C and bortezomib deregulated other biological processes including ER stress, protein folding, centrosome and mitotic spindle apparatus, carcinogen metabolism, metabolic pathways and cytoskeletal regulators (Supplementary Table S2). Representative target genes (e.g., *DDIT3*, *HSPA6* and *CENPF*) from each of these processes were validated by qRT–PCR with the majority of them demonstrating regulation as determined by microarray analysis (Figure 5C and Supplementary Figure S1A).

Overall, we found that co-treatment with I3C and bortezomib causes widespread gene deregulation that impinges on multiple pathways ultimately resulting in cell death (Figure 5D). A summary of target genes both related to and identified by microarray analysis were confirmed by qRT–PCR and western analysis (Supplementary Table S3).

### I3C and bortezomib co-treatment inhibits the growth of OVCAR5 tumour xenografts in nude mice

**Figure 6:**
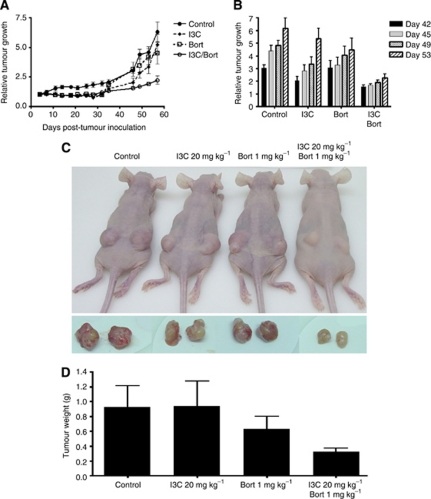
Indole-3-carbinol and bortezomib co-treatment inhibit human ovarian tumour xenografts in nude mice. (**A**) Relative tumour growth of OVCAR5 xenografts treated with vehicle (control), 20 mg kg^−1^ I3C, 1 mg kg^−1^ bortezomib or in combination measured from 0 to 53 days post treatment or (**B**) measured 42, 45, 49 and 53 days post treatment. The data shown represent the mean±s.e.m. (*n*=8). (**C**) Representative tumour images of control- (vehicle), I3C- and bortezomib-treated mice pre and post dissection, with corresponding (**D**) tumour weight 53 days post treatment. The data shown represent the mean±s.e.m. (*n*=8).

To examine the effect of I3C and bortezomib *in vivo*, we monitored the tumour growth of OVCAR5 tumour xenografts in nude mice treated with I3C and/or bortezomib. Initially, we performed a dose-finding study to establish the tolerable dosages of I3C and bortezomib (see Supplementary Methods for details and Supplementary Figure S2). Based on our dose-finding data, we randomly assigned mice to the following four treatment groups: vehicle (control); I3C (20 mg kg^−1^); bortezomib (1 mg kg^−1^) or combination treatment (20 mg kg^−1^ I3C with 1 mg kg^−1^ bortezomib). Although treatment with I3C or bortezomib alone initially induced tumour regression (Figure 6A), these mice relapsed after prolonged treatment (⩾31 days post treatment). In contrast, the combination of I3C and bortezomib significantly inhibited tumour growth (Figure 6A) compared with control animals (64.6% tumour reduction, *P*<0.001) or individual treatment with I3C (47.6% tumour reduction, *P*=0.007) or bortezomib (35.9% tumour reduction, *P*=0.029) by the final day of treatment, consistent with our *in vitro* results. Indeed, the final weight (65.4% tumour reduction in I3C/bortezomib combination *vs* control, *P*=0.053) and appearance of the tumours post treatment were consistent with the measurements obtained from earlier time points (Figures 6B–D). Moreover, we found that co-treatment with reduced bortezomib levels (0.5 mg kg^−1^) inhibited tumour growth and increasing levels of I3C significantly potentiated bortezomib-induced tumour regression in a dose-dependent manner, emphasising the synergistic effect of these two drugs (Supplementary Figure S2).

## Discussion

Ovarian cancer is a highly heterogeneous disease involving the deregulation of multiple genes and pathways commonly evaded by gene- or pathway-specific agents (Donninger *et al*, 2004; Landen *et al*, 2008). We rationalised that targeted therapeutics that can overcome drug resistance and exploit pathways involved in tumourigenesis may be beneficial in the treatment of ovarian cancer. Here, we have shown that the combination of potent anticarcinogens, I3C and bortezomib, inhibits several signalling pathways and exerts antiproliferative and apoptotic effects in ovarian cancer cells, consistent with the individual properties of these drugs. Microarray analysis and validation studies showed that combination I3C and bortezomib induces pleiotropic effects reflecting its use as a multi-targeted combination therapy. We have extended the limited data of I3C treatment in ovarian cancer and found that I3C and bortezomib combination demonstrate synergistic cytotoxicity through additionally influencing chemoresistance, metastasis, cytoskeletal regulation, ER stress, carcinogen metabolism and metabolic pathways. Importantly, we show that I3C and bortezomib co-treatment inhibits tumour growth *in vivo*. Collectively, these data provide the first evidence that combining I3C with bortezomib is effective and may demonstrate a clinical benefit in the management of ovarian cancer.

In our study, we found that inhibition of carcinogenesis-associated pathways was the main mechanism of combination I3C/bortezomib-induced cytotoxicity, particularly through inhibition of cell proliferation, DNA replication and promotion of cell cycle arrest, consistent with our findings *in vivo*. Additionally, deregulation of the cell cycle was extended to centrosome and mitotic spindle apparatus inactivation. As many of the target genes altered by combination I3C and bortezomib were involved in cell cycle control, we explored whether the mechanism of these drugs may act through a common cell-cycle-related factor. Given that I3C and bortezomib inhibit the cyclin E pathway using independent mechanisms (Nguyen *et al*, 2008; Uddin *et al*, 2008, 2009; Bruning *et al*, 2009), we considered whether overexpression of cyclin E would cause an enhanced response to combination therapy. However, synergy was more robust in low-cyclin-E-expressing OVCAR5 cells compared with OVCAR3 cells, implying that the mechanism of I3C and bortezomib is cyclin-E-independent, and that other biological pathways are responsible for the effects seen with this drug combination.

Indeed, I3C and bortezomib combination induced apoptosis and inhibited several signal transduction pathways that promote cell survival, including MAPK, TGF*β*, NFkB and PI3K-AKT. Co-treatment with these two agents also affected metastasis and tumour suppressor genes (TSGs). One metastasis-associated gene, *MET*, is a proto-oncogene that was downregulated both at the RNA and protein level consistent with our microarray analysis. MET promotes tumour growth, angiogenesis and activates multiple cell survival signalling pathways (Xiao *et al*, 2001; Birchmeier *et al*, 2003; Derksen *et al*, 2003; Garcia *et al*, 2007). Aberrant MET activation occurs in many human cancers, correlates with poor prognosis, and is considered an important candidate for targeted therapy (Kaposi-Novak *et al*, 2006; Comoglio *et al*, 2008; Liu *et al*, 2008). However, we also identified and confirmed the upregulation of SNAI1, which promotes tumour growth and metastasis in various solid tumours, including ovarian (Jin *et al*, 2010). Several TSGs were deregulated including *CCBE1*. Interestingly, *CCBE1* is a new TSG candidate that was upregulated in our microarray dataset and is frequently inactivated in ovarian cancer (Barton *et al*, 2010). Several biotransformation enzymes required for carcinogen detoxification were upregulated in our microarray dataset similar to treatment with I3C in prostate cancer cells (Li *et al*, 2003). Metabolic enzymes required for post-translational modifications were deregulated, including downregulation of *PIGM*, a gene encoding a glycosylphosphatidylinositol biosynthetic enzyme required for growth in yeast (Maeda *et al*, 2001; Kim *et al*, 2007). Among the validated targets, ER stress (*DDIT3* and *ATF3*) and heat shock proteins (*HSPA6*) were the most severely upregulated. This is typically observed in cells undergoing cellular stress (Santoro, 2000), and is preceded by ER stress and DDIT3-induced apoptosis (Zimmermann *et al*, 2000; Fribley and Wang, 2006). We also found that chemoresistance-associated genes *TOP2A* (cell-cycle-regulated gene) and *ABCC4* (multidrug-resistant gene) were both downregulated, although the latter was just above the log-fold threshold (−1.47). TOP2A is upregulated in chemoresistant ovarian and hepatocellular tumours, and correlates with reduced survival (Ju *et al*, 2009; Wong *et al*, 2009), implying that I3C/bortezomib treatment may circumvent drug resistance. Consistent with this, our data also demonstrated that I3C/bortezomib treatment sensitised cells to standard platinum-based chemotherapeutic agents.

From a clinical perspective, these findings in conjunction with our I3C/bortezomib-induced tumour regression data provide compelling evidence for the potential treatment of patients that succumb to platinum-resistant disease. Future studies that address whether I3C/bortezomib treatment can potentiate the effects of cisplatin and carboplatin *in vivo* or additional *in vivo* studies that compare I3C/bortezomib treatment with standard combination chemotherapy (e.g., cisplatin/paclitaxel) could highlight the advantage of this novel drug combination.

We acknowledge several limitations of our study. First, we selected a single cell line and time point to determine the mechanism of I3C and bortezomib action. Overlap of the target genes from an additional OVCAR3 microarray study may have narrowed down the most essential target genes/pathways required for I3C and bortezomib action. As the cytotoxic effect of I3C and bortezomib was rapid (24 h), a compilation of gene expression profiles from earlier time points may provide more insight into the mechanism of these combined agents in ovarian cancer, similar to the study performed with I3C in prostate cancer cells at three time points (Li *et al*, 2003). Second, we primarily focused on target genes that had log-fold changes >1.5 or <−1.5. According to our arbitrary cutoff, 189 genes were significantly deregulated in our microarray analysis that failed to meet the log-fold ratio criteria. This suggests that additional target genes/pathways that confer subtle changes in gene expression may contribute to the deleterious effects of I3C and bortezomib. Third, bortezomib resistance is a significant problem despite clinical success in patients with myeloma and mantle cell lymphoma (Ruschak *et al*, 2011). Perhaps using alternative, irreversible proteasome inhibitors in combination with I3C may demonstrate comparable or improved effects.

In summary, this is the first study to describe the synergistic effects of combination I3C and bortezomib in ovarian cancer. The ability of this combination of agents to inhibit tumour growth *in vivo*, sensitise cells to standard chemotherapeutic agents, promote apoptosis and interfere with genes affecting multiple pathways important for cancer progression supports the utility of I3C and bortezomib in the treatment of ovarian cancer.
