# Supplementary material for: Indole-3-carbinol synergistically sensitises ovarian cancer cells to bortezomib treatment
Source: Br J Cancer. 2011 Dec 13;106(2):333–43. doi: 10.1038/bjc.2011.546 (PMC3261668; doi:10.1038/bjc.2011.546)
Supplement: Supplementary Figure S1 [file bjc2011546x1.ppt]

## Slide 1
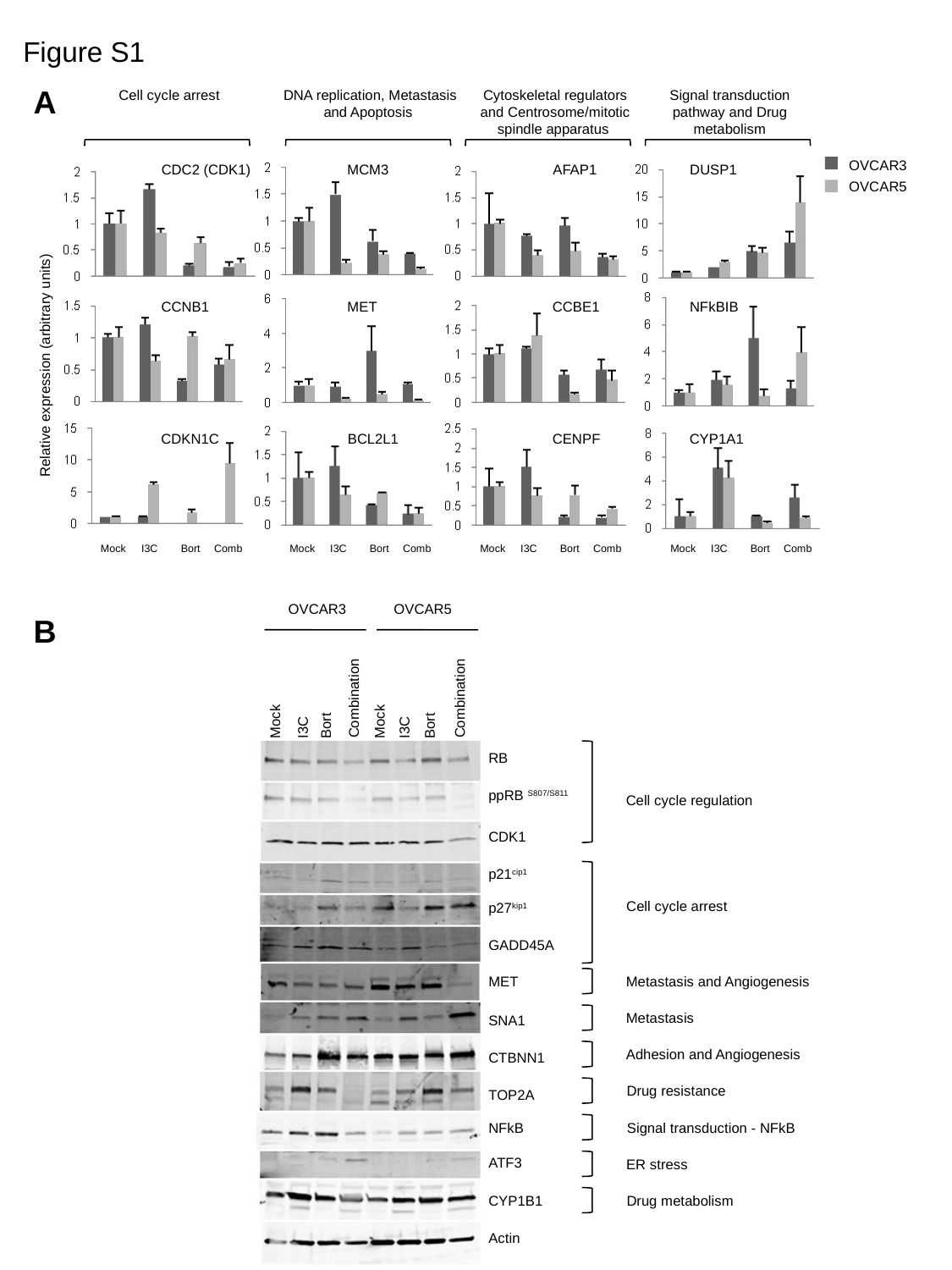

Figure S1
A
Cell cycle arrest
DNA replication, Metastasis and Apoptosis
Cytoskeletal regulators and Centrosome/mitotic spindle apparatus
Signal transduction pathway and Drug metabolism
OVCAR3
OVCAR5
CDC2 (CDK1)
MCM3
AFAP1
DUSP1
CCNB1
MET
CCBE1
NFkBIB
Relative expression (arbitrary units)
CDKN1C
BCL2L1
CENPF
CYP1A1
Mock
I3C
Bort
Comb
Mock
I3C
Bort
Comb
Mock
I3C
Bort
Comb
Mock
I3C
Bort
Comb
OVCAR3
OVCAR5
B
Combination
Combination
Mock
Mock
Bort
Bort
I3C
I3C
RB
ppRB S807/S811
Cell cycle regulation
CDK1
p21cip1
Cell cycle arrest
p27kip1
GADD45A
MET
Metastasis and Angiogenesis
Metastasis
SNA1
Adhesion and Angiogenesis
CTBNN1
Drug resistance
TOP2A
Signal transduction - NFkB
NFkB
ATF3
ER stress
CYP1B1
Drug metabolism
Actin
