# Supplementary material for: Indole-3-carbinol synergistically sensitises ovarian cancer cells to bortezomib treatment
Source: Br J Cancer. 2011 Dec 13;106(2):333–43. doi: 10.1038/bjc.2011.546 (PMC3261668; doi:10.1038/bjc.2011.546)
Supplement: Supplementary Figure S2 [file bjc2011546x2.ppt]

## Slide 1
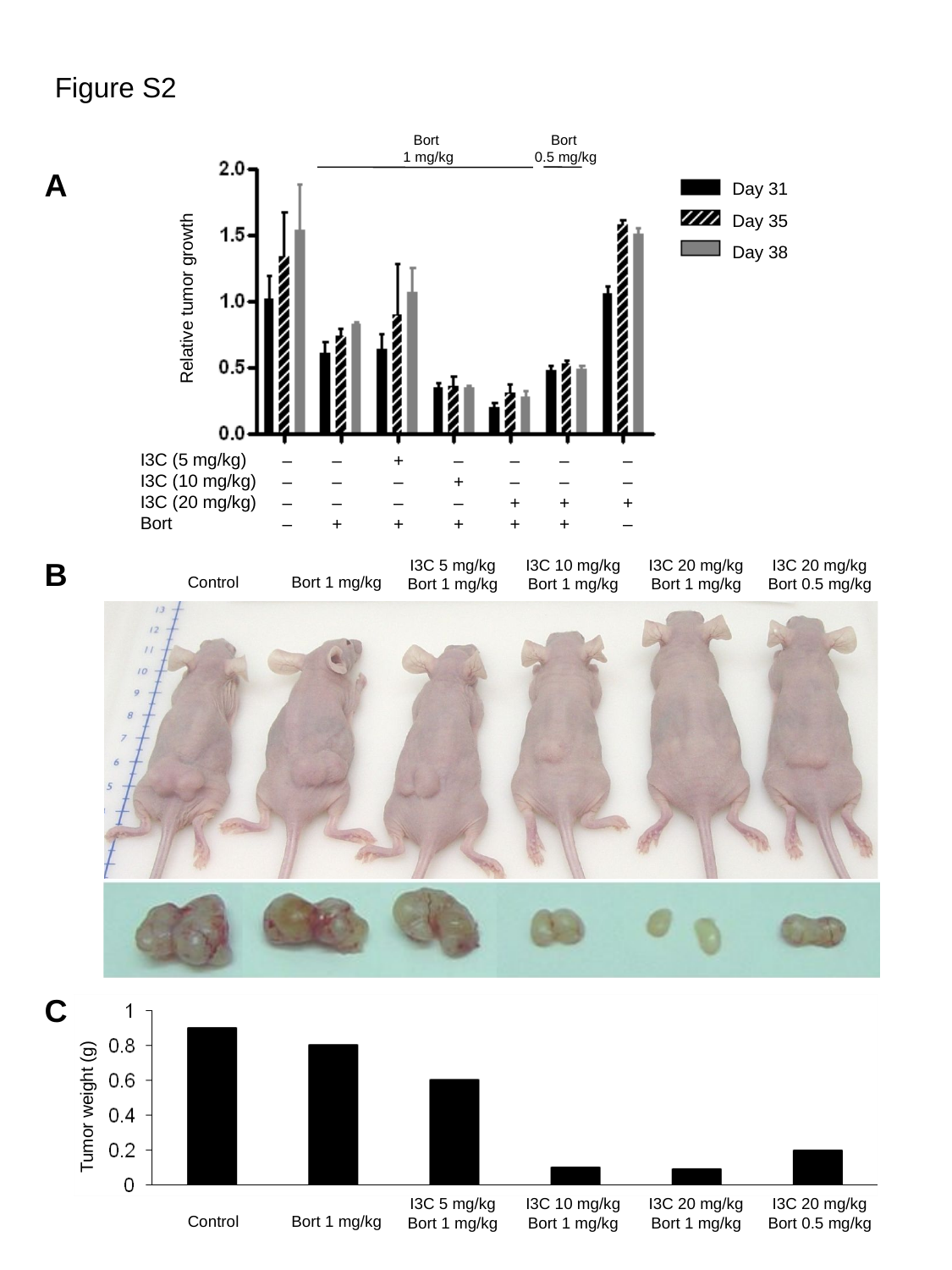

Figure S2
Bort
1 mg/kg
Bort
0.5 mg/kg
A
Day 31
Day 35
Day 38
Relative tumor growth
I3C (5 mg/kg)
I3C (10 mg/kg)
I3C (20 mg/kg)
Bort
–
–
–
–
–
–
–
+
+
–
–
+
–
+
–
+
–
–
+
+
–
–
+
+
–
–
+
–
B
I3C 5 mg/kg
Bort 1 mg/kg
I3C 10 mg/kg
Bort 1 mg/kg
I3C 20 mg/kg
Bort 1 mg/kg
I3C 20 mg/kg
Bort 0.5 mg/kg
Control
Bort 1 mg/kg
C
Tumor weight (g)
I3C 5 mg/kg
Bort 1 mg/kg
I3C 10 mg/kg
Bort 1 mg/kg
I3C 20 mg/kg
Bort 1 mg/kg
I3C 20 mg/kg
Bort 0.5 mg/kg
Control
Bort 1 mg/kg
