# Supplementary material for: Indole-3-carbinol synergistically sensitises ovarian cancer cells to bortezomib treatment
Source: Br J Cancer. 2011 Dec 13;106(2):333–43. doi: 10.1038/bjc.2011.546 (PMC3261668; doi:10.1038/bjc.2011.546)
Supplement: Supplementary Table S1 [file bjc2011546x3.doc]

Table S1. Primer sequences used for qRT-PCR.

| Gene Symbol | Forward sequence | Reverse sequence |
| --- | --- | --- |
| *CDKN1A* | CATTGTGGGAGGAGCTGTGA | ATGTGTCCTGGTTCCCGTTTC |
| *CCNB1* | TTGGGGACATTGGTAACAAAGTC | ATAGGCTCAGGCGAAAGTTTTT |
| *CCNE1* | TGAAGTGTGAGTGCCTCTGG | AGGGGACTTAAACGCCACTT |
| *CDC2* | AATCTATGATCCAGCCAAACGAA | TCTTAATCTGATTGTCCAAATCATTAAAA |
| *GADD45A* | GAGAGCAGAAGACCGAAAGGA | CACAACACCACGTTATCGGG |
| *GADD45B* | TTTGTTTGTGGCAGCAACTC | TCGGATTTTGCAATTTCTCC |
| *DDIT3* | AAGGCACTGAGCGTATCATGT | TGAAGATACACTTCCTTCTTGAACA |
| *HSPA6* | CAAGGTGCGCGTATGCTAC | GCTCATTGATGATCCGCAACAC |
| *ATF3* | TCCTCTGCGCTGGAATCAG | GGCCGATGAAGGTTGAGCA |
| *JUN* | GCCGGTCTACGCAAACCTC | GGACTCCATGTCGATGGGG |
| *BAG3* | GGCCCCAAGGAGACTCCAT | GCCTCAGTTCGGAATCGCT |
| *BCL10* | CTTGTTGAATCTATTCGGCGAGA | TGGAAAAGGTTCACAACTGCT |
| *TOP2A* | TCATCAAGATTGTGGGTCTTCAG | CCTCCAGAAAACGATGTCGCA |
| *ABCC4* | ATCCAGACATTGCTACAAGTGG | GGTATTGCGATCCAAGGAATCAC |
| *CYP1A1* | CACCCTCATCAGTAATGGTCAGA | AACGTGCTTATCAGGACCTCA |
| *CHST4* | CCTGCTGTTTCTGGTTTCCCA | GCCCCACAAAAGAAGAGCCA |
| *PIGM* | CTGAGAGCCACGTACCGTTAC | AAAGACACAGTAGCCACAAGC |
| *CCBE1* | CGACTAAATACCCGTGTCTGAAG | TCGGCACAAACGTCGTAATCT |
| *CENPF* | GAGATGGAGTCCAAGTTGGCG | TGGGTTTCACTGCACCAGTC |
| *AFAP1* | AGCAGCTCTTATGAGTCGTATGA | ACAGCAGTTTGGTGTCTTTGA |
| *BCL2L1* | GGTCGCATTGTGGCCTTTTTC | TGCTGCATTGTTCCCATAGAG |
| *CYP1B1* | AAGTTCTTGAGGCACTGCGAA | CCGGTACGTTCTCCAAATCC |
| *DUSP1* | CCAGTACAAGAGCATCCCTGT | AGTGGACAAACACCCTTCCTC |
| *NFKBIB* | GTCGCGTGCTTGGGAAAAG | CCAGGAAGGGTTCATGCTGAT |
| *BCL2* | GAACTGGGGGAGGATTGTGG | CCGGTTCAGGTACTCAGTCA |
| *ATF4* | GACCACGTTGGATGACACTTG | GGGAAGAGGTTGTAAGAAGGTG |
| *ATF6* | CAGCCTACTGTGGTACAACTTC | GGACAGGTTTAGTCACGGAAAG |
| *MAX* | GGAGAGCGACGCTGACAAA | GTCGTCAATATCTTGCTGGTGT |
| *CDKN1C* | ACATCCACGATGGAGCGTC | GGAAGTCGTAATCCCAGCGG |
| *MET* | TGGTGCAGAGGAGCAATGG | CCCAGTCTTGTACTCAGCAAC |
| *MCM3* | TGTTCTCTAGTTCGTCCCAAAGT | TTGGTAGGATAGACAGAGCTGG |
| *RASSF6* | GCTCACCAGTACCCCTCTTG | GCTGTATAGGTCGTTTTACTCCC |
| *GAPDH* | ATGGGGAAGGTGAAGGTCG | GGGGTCATTGATGGCAACAATA |
