# Supplementary material for: Indole-3-carbinol synergistically sensitises ovarian cancer cells to bortezomib treatment
Source: Br J Cancer. 2011 Dec 13;106(2):333–43. doi: 10.1038/bjc.2011.546 (PMC3261668; doi:10.1038/bjc.2011.546)
Supplement: Supplementary Table S3 [file bjc2011546x5.doc]

Table S3. Summary of target genes differentially expressed in OVCAR5 cells cotreated with I3C and bortezomib classified by pathway and validated by qRT-PCR and/or Western in both OVCAR3 and OVCAR5 cells.

|  |  |  | **Validation** | |
| --- | --- | --- | --- | --- |
| **Gene Target** | **Microarray**  **Discovery** | **Pathway** | **qRT-PCR** | **Western** |
| CDC2 (CDK1) | Y (-2.07) | Cancer - Cell cycle regulation | V | V |
| CCNB1 | Y (-1.23) | Cancer - Cell cycle regulation | V | ND |
| CCNE1 | N | Cancer - Cell cycle regulation | V | ND |
| RB and ppRB^S807/811^ | N | Cancer - Cell cycle regulation | ND | V |
| CDKN1A (p21^cip1^) | Y (+3.10) | Cancer - Cell cycle arrest | V | V |
| CDKN1B (p27^kip1^) | N | Cancer - Cell cycle arrest | ND | V |
| CDKN1C (p57^kip2^) | Y (+2.07) | Cancer - Cell cycle arrest | V | ND |
| GADD45A | Y (+3.21) | Cancer - Cell cycle arrest | V | V |
| GADD45B | Y (+2.42) | Cancer - Cell cycle arrest | V | ND |
| MCM3 | Y (-1.82) | Cancer - DNA replication | V | ND |
| JUN | Y (+2.44) | Cancer - Signal transduction/ MAPK | V | ND |
| DUSP1 | Y (+3.07) | Cancer - Signal transduction/ MAPK | V | ND |
| MAX | N | Cancer - Signal transduction/ MAPK | V | ND |
| NFkBIB | Y (+1.73) | Cancer - Signal transduction/ NFkB | V | ND |
| NFkB | N | Cancer - Signal transduction/ NFkB | ND | V |
| BCL2 | N | Cancer - Apoptosis | V | ND |
| BCL10 | Y (+2.23) | Cancer - Apoptosis | V | ND |
| BAG3 | Y (+2.40) | Cancer - Apoptosis | V | ND |
| BCL2L1 | Y (-1.51) | Cancer - Apoptosis | V | ND |
| RASSF6 | Y (+1.81) | Cancer - Apoptosis | V | ND |
| MET | Y (-1.63) | Cancer - Metastasis and angiogenesis | V | V |
| SNAI1 | Y (1.81) | Cancer - Metastasis | ND | V |
| CTNNB1 | N | Cancer - Adhesion and angiogenesis | ND | V |
| TOP2A | Y (-2.93) | Cancer - Drug resistance | V | V |
| ABCC4 | Y (-1.47) | Cancer - Drug resistance | V | ND |
| DDIT3 (CHOP) | Y (+5.03) | ER stress | V | ND |
| ATF3 | Y (+4.61) | ER stress | V | V |
| ATF4 | N | ER stress | V | ND |
| ATF6 | N | ER stress | V | ND |
| HSPA6 | Y (+7.17) | Heat shock | V | ND |
| CYP1A1 | Y (+2.47) | Drug metabolism | V | ND |
| CYP1B1 | Y (+2.23) | Drug metabolism | V | V |
| CHST4 | Y (-2.14) | Glycosaminoglycan biosynthesis | V | ND |
| PIGM | Y (-1.91) | Glycosylphosphatidylinositol biosynthesis | V | ND |
| CENPF | Y (-2.46) | Centrosome/mitotic spindle apparatus | V | ND |
| AFAP1 | Y (-2.35) | Cytoskeletal regulator | V | ND |
| CCBE1 | Y (+2.21) | Cytoskeletal regulator | V | ND |
