# Supplementary material for: Indole-3-carbinol synergistically sensitises ovarian cancer cells to bortezomib treatment
Source: Br J Cancer. 2011 Dec 13;106(2):333–43. doi: 10.1038/bjc.2011.546 (PMC3261668; doi:10.1038/bjc.2011.546)
Supplement: Supplementary Figures and Tables Legend [file bjc2011546x6.doc]

**SUPPLEMENTARY FIGURE LEGENDS**

**Figure S1**. I3C and bortezomib inhibit carcinogenesis, deregulate enzymes required for carcinogen metabolism and downregulate mitotic spindle apparatus, centrosome and cytoskeletal regulators in OVCAR3 and OVCAR5 cells. OVCAR3 cells were treated with vehicle (mock), 270 µM I3C, 18.8 nM bortezomib or in combination for 24 h. OVCAR5 cells were treated at equipotent I3C (675 µM) and bortezomib (37.5 nM) concentrations. **A**, RNA was isolated and qRT-PCR of candidate target genes identified from microarray analysis was performed in OVCAR3 and OVCAR5 cells. Target genes are categorized by function. **B**, Whole cell extracts were isolated and immunoblotted with the indicated antibodies categorized by function. Actin was used as a loading control.

**Figure S2**. I3C and bortezomib co-treatment inhibits the growth of human ovarian tumor xenografts in a dose-dependent manner in nude mice. **A**, Relative tumor growth of OVCAR5 xenografts measured 31, 35 and 38 d post-treatment. The data shown represent the mean ± SEM (n=2). **B**, Representative tumor images of control- (vehicle), I3C- and bortezomib-treated mice pre- and post-dissection with corresponding **C**, tumor weight after 53 d post-treatment. The data shown represent the mean.

**Table S1**. Primer sequences used for qRT-PCR are listed. Primers were designed using PrimerBank (http://pga.mgh.harvard.edu/primerbank/index.html).

**Table S2**. Classification and fold changes of genes with differential expression in OVCAR5 cells co-treated with 675 µM I3C and 37.5 nM bortezomib for 24 h. Differentially expressed genes with log-fold changes >1.5 (upregulated) or <-1.5 (downregulated) (*P*<0.0025) compared to control (mock) are listed and further classified by function.

**Table S3**. Summary of validated target genes differentially expressed in OVCAR5 cells co-treated with I3C and bortezomib and classified by function. OVCAR3 cells were treated with vehicle (mock), 270 µM I3C, 18.8 nM bortezomib or in combination for 24 h. OVCAR5 cells were treated at equipotent I3C (675 µM) and bortezomib (37.5 nM) concentrations. Nine non-microarray and 28 microarray representative target genes were validated by qRT-PCR and/or Western in both OVCAR3 and OVCAR5 cells. Y, positive for differential expression; V, validated target gene; N, not identified by microarray analysis; ND, not determined.
