# Supplementary material for: Indole-3-carbinol synergistically sensitises ovarian cancer cells to bortezomib treatment
Source: Br J Cancer. 2011 Dec 13;106(2):333–43. doi: 10.1038/bjc.2011.546 (PMC3261668; doi:10.1038/bjc.2011.546)
Supplement: Supplementary Methods [file bjc2011546x7.doc]

**SUPPLEMENTARY METHODS**

**Dose-Finding *In Vivo* Tumor Xenograft Studies**

Six-week-old female nude mice were obtained from Charles River Laboratories (Wilmington, MA) and maintained according to IACUC guidelines. Mice were inoculated subcutaneously in both flanks with an equal volume of 8 x 10^6^ OVCAR5 cells and matrigel (Becton Dickinson) in a total volume of 200 µl. Initially, mice were randomly divided into 4 treatment groups with 5 mice per group (n=10). Treatments were as follows: vehicle (control); I3C alone (50 mg/kg); bortezomib alone (1 mg/kg) and the drug combination (50 mg/kg I3C with 1 mg/kg bortezomib). However, we found that the drug combination was toxic. As a result, we modified the conditions by lowering the drug dosages 10 d post-treatment. The 5 control mice were then divided into a range of I3C dosage treatments: I3C alone (5, 10 and 20 mg/kg in sesame seed oil/PBS) and vehicle control. Similarly, the 5 bortezomib-treated mice were divided into a range of I3C/bortezomib combination treatment groups: 5 mg/kg I3C with 1 mg/kg bortezomib; 10 mg/kg I3C with 1 mg/kg bortezomib; 20 mg/kg I3C with 1 mg/kg bortezomib; 20 mg/kg I3C with 0.5 mg/kg bortezomib and bortezomib alone (1 mg/kg). Treatment was given intraperitoneally twice weekly starting 4 d post-inoculation. Tumor size was measured twice weekly with a caliper and tumor volume was calculated as follows: L x W^2^, where L = length and W = width. Data were expressed relative to the initial tumor volume 4 d post-inoculation. The initial tumor volume was set to 1 for each treatment group.
